# Frailty in Acute and Chronic Coronary Syndrome Patients Entering Cardiac Rehabilitation

**DOI:** 10.3390/jcm10081696

**Published:** 2021-04-15

**Authors:** Francesco Giallauria, Anna Di Lorenzo, Elio Venturini, Mario Pacileo, Antonello D’Andrea, Umberto Garofalo, Felice De Lucia, Crescenzo Testa, Gianluigi Cuomo, Gabriella Iannuzzo, Marco Gentile, Cinzia Nugara, Filippo M Sarullo, Nastasia Marinus, Dominique Hansen, Carlo Vigorito

**Affiliations:** 1 Department of Translational Medical Sciences, “Federico II” University of Naples, 80131 Naples, Italy; dilorenzoanna2@gmail.com (A.D.L.); umberto.garofalo94@gmail.com (U.G.); felice.delucia@libero.it (F.D.L.); kre.testa@gmail.com (C.T.); gianluigi.cuomo95@gmail.com (G.C.); vigorito@unina.it (C.V.); 2 Cardiac Rehabilitation Unit, Azienda USL Toscana Nord-Ovest, Cecina Civil Hospital, 57023 Cecina (LI), Italy; vent.elio@tin.it; 3 Division of Cardiology/UTIC, “Umberto I” Hospital, Nocera Inferiore (ASL Salerno), 84014 Nocera Inferiore (SA), Italy; pacmario@yahoo.it (M.P.); antonellodandrea@libero.it (A.D.); 4 Division of Cardiology, “Luigi Vanvitelli” University of Naples, 80131 Naples, Italy; 5 Department of Clinical Medicine and Surgery, “Federico II” University of Naples, 80131 Naples, Italy; gabriella.iannuzzo@unina.it (G.I.); margenti@unina.it (M.G.); 6 Cardiovascular Rehabilitation Unit, Buccheri La Ferla Fatebenefratelli Hospital, 90123 Palermo, Italy; cinzianugara@gmail.com (C.N.); fsarullo@neomedia.it (F.M.S.); 7 REVAL-Rehabilitation Research Center, Faculty of Rehabilitation Sciences, Hasselt University, BE3590 Diepenbeek, Belgium; nastasia.marinus@uhasselt.be (N.M.); dominique.hansen@uhasselt.be (D.H.); 8 BIOMED-Biomedical Research Center, Hasselt University, BE3590 Diepenbeek, Belgium; 9 Heart Centre Hasselt, Jessa Hospital, BE3500 Hasselt, Belgium

**Keywords:** frailty, cardiovascular disease, cardiac rehabilitation, acute coronary syndrome, chronic coronary syndrome, exercise training

## Abstract

Worldwide population ageing is partly due to advanced standard of care, leading to increased incidence and prevalence of geriatric syndromes such as frailty and disability. Hence, the age at the onset of acute coronary syndromes (ACS) keeps growing as well. Moreover, ageing is a risk factor for both frailty and cardiovascular disease (CVD). Frailty and CVD in the elderly share pathophysiological mechanisms and associated conditions, such as malnutrition, sarcopenia, anemia, polypharmacy and both increased bleeding/thrombotic risk, leading to a negative impact on outcomes. In geriatric populations ACS is associated with an increased frailty degree that has a negative effect on re-hospitalization and mortality outcomes. Frail elderly patients are increasingly referred to cardiac rehabilitation (CR) programs after ACS; however, plans of care must be tailored on individual’s clinical complexity in terms of functional capacity, nutritional status and comorbidities, cognitive status, socio-economic support. Completing rehabilitative intervention with a reduced frailty degree, disability prevention, improvement in functional state and quality of life and reduction of re-hospitalization are the goals of CR program. Tools for detecting frailty and guidelines for management of frail elderly patients post-ACS are still debated. This review focused on the need of an early identification of frail patients in elderly with ACS and at elaborating personalized plans of care and secondary prevention in CR setting.

## 1. Introduction

Older population keeps growing contributing to the increase in the overall population size, also strongly influenced by major declines in leading causes of mortality. These demographic changes reverberate in society, increasing healthcare needs and costs, which are expected to steeply increase in the years to come [1]. The proportion of people aged over 65 years will pass 25% by 2030 and the number of elderly people will double in Europe from 87.5 million in 2010 to 152.6 million in 2060 [2]. This aging process will give more prominence to geriatric syndromes and, in particular, to the concept of frailty.

Acute coronary syndrome (ACS) is one of the leading CAD presentations. It is estimated that over half of all people hospitalized for acute coronary syndrome (ACS) are elderly patients and at least 10% of them aged 65 years or older are considered frail [3]. Although frailty is better described and characterized in patients with heart failure or valvular heart disease; frailty detection has not yet entered in routine ACS patient assessment in the elderly and guidelines for early identification of frailty with appropriate tools in post-ACS are still unclear and unsatisfactory.

The present paper therefore aims to review the most relevant data emphasizing the need for an early identification of pre-frailty and frailty in CAD-ACS patients and the elaboration of personalized plans of care and secondary prevention in cardiac rehabilitation.

## 2. Frailty: Definition and Models

In the past decades, many definitions of frailty have been proposed; many of them identify frailty as an ageing-related loss or reduction of the ability to react to stressors or events, considering frailty not as a “disease”, but as a high-risk condition for developing acute and chronic comorbidity and disability [4]. Frailty is a multifactorial condition, arising from different pathophysiological mechanisms, including inflammation, hypothalamic-hypophysis axis and anabolic-catabolic hormone imbalance [5,6,7,8].

In the last decades, two main models have been proposed for diagnosing frailty [9,10]: the phenotype model proposed by Fried et al. [9] describes frailty with five physical items: (1) unintentional weight loss at least 4.5 Kg in the past year or ≥5% of body weight in the prior year; (2) self-reported exhaustion identified by two questions from CES-D depression scale [11]; (3) reduced handgrip strength stratified for gender and body mass index (BMI); (4) slow gait speed stratified for gender and height and (5) low physical activity-related energy expenditure based on participant self-report by Minnesota Leisure Time Physical Activity Questionnaire (MTLAQ-short version) [12]. The absence of any of these criteria define “robust” patients; “pre-frail” patients have one or two and “frail” patients have three or more of these criteria [9].

The Canadian Study of Health and Aging Frailty Index (FI), proposed by Rockwood et al. [4,13,14], is based on a cumulative deficit model, comprising 70 items including clinical signs, symptoms, diseases and comorbidities to build a FI score, calculating the proportion of the individual deficits relative to the total considered in the model. The cumulative model requires a minimum of 30 explored items and gives as result a number between 0 and 1. Many other tools derived from these two main models have been described and applied in cardiac rehabilitation setting [15,16].

Particularly in elderly patients, currently, one of the best models considered to evaluate frailty is the Frailty Index derived from the Comprehensive Geriatric Assessment (CGA) [17]. FI-CGA consists of a multidimensional assessment of different health domains in elderly patients, exploring 10 domains (cognition, motivation, disability, communication, mobility, balance, bowel/bladder function, nutrition, social ability and comorbidity) to build a single domain score or to categorize patients in three classes of frailty: mild (0–7 FI-CGA), moderate (7–13 FI-CGA) and severe (>13 FI-CGA) [18].

Recently, Liguori et al. [19] proposed a quick tool to identify multidimensional frailty in the elderly derived from the Italian Frailty index (IFi), an Italian modified version of Rockwood’s Frailty index, named AGILE (a 10-item tool created starting from the more predictive items of the four domains of frailty investigated by IFi (mental, physical, socioeconomic and nutritional) [19]. Main domains and tools for frailty evaluation are reported in Table 1.

Hence, these frailty assessment tools should be used very often in cardiovascular medicine. Cardiovascular diseases have a crucial impact on the global health stage, especially in the aging population [1]; recent data showed that in the US, 53% of the patients hospitalized for Non-ST-Elevation-Acute Coronary syndrome (NSTE-ACS) are over 75 years and 35.1% hospitalized for ST-Elevation-Acute Coronary syndrome (STE-ACS) are over 75 years [20].

The Italian registries BLITZ-1 and BLITZ-2 also reported that 27% of ACS patients were over 75 years, of NSTEMI patients 28% of the patients were over 75 years. [21,22]. Oltrona et al. [23], who studied 1773 patients with ACS requiring Cardiac Intensive Care Unit, reported that more than half of the patients were over 70 years. Moreover, ACS in elderly patients has a more severe clinical phenotype: non-cardiac comorbidities are most common in elderly patients at the onset of the acute coronary event; 84% of death for coronary artery disease in US occurs in patients aged 65 or older [24].

## 3. Chronic Coronary Syndrome and Frailty in Elderly Patients

Atherosclerosis, calcification and tortuosity of coronary artery are a hallmark of aging; notably, coronary artery disease is still the leading cause of morbidity and mortality in elderly patients [25,26]. Quayyum et al. [27] reported a frailty prevalence of around 28% according to the Fried phenotype model in a population aged >80 years with coronary artery disease; survival curves for frail and pre-frail patients showed a high short-term mortality in pre-frail patients and a greater mortality in frail patients. Moreover, frailty seemed to negatively impact the quality of life, independently from the NHYA class. Moreover, the overlap between frailty, comorbidity and disability (around 20%) suggest they are co-working in health status worsening.

**Figure 1:**
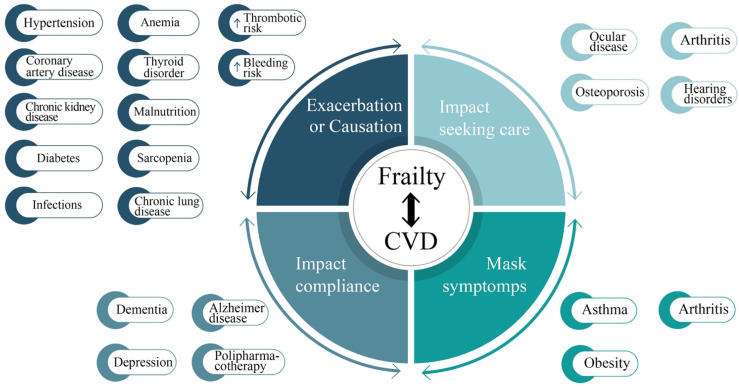
Principal comorbidity overlapping frailty and cardiovascular disease in elderly patients. CVD, cardiovascular disease; 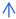, increase of.

The risk of cardiovascular disease onset increased in frail and pre-frail patients aged >65 years. [28]. Coexistence of frailty and cardiovascular disease (CVD) impact on patient’s prognosis of each of the two conditions [29] that worsen prognosis with higher risk of short-term mortality (3 months) [30]. According to Frailty Phenotype model the prevalence of Cardiovascular Disease (CVD) resulted in a two- or threefold increase in frail patients and a trend to increase in pre-frailty; moreover, it has been reported an increase of prevalence of subclinical cardiovascular “abnormalities” detected with noninvasive testing: left ventricular hypertrophy, wall motion impairment at echocardiography, systolic hypertension, ECG abnormalities, increased carotid intima-media thickness and ankle arm index <0.8, RMN evidence of stroke [31]. In hospitalized elderly patients, malnutrition and hypoalbuminemia can indicate either a frailty status or a consequence of advanced CAD. Reasons can be found in similarity and overlapping of biologic and pathophysiologic mechanisms leading to both frailty and cardiovascular disease, such as inflammatory status and age-related subclinical cardiovascular alteration (Figure 1) [32,33].

In chronic coronary syndrome patients heart failure (HF) is strongly prevalent in the elderly population and the risk of frailty is 3.4 times higher in HF patients with a large prevalence of pre-frailty (46%) and frailty (40%) [34]. Chronic inflammation may be the pathophysiological link between HF and sarcopenia [35]. In addition, chronic HF leads to functional impairment, a slow gait speed, balance disorders and disability. Interestingly, HF patients often experience malabsorption with consequent loss of body weight and macro- and micro-nutrients deficiency (i.e., iron deficiency resulting in anemia and consequently worsening of heart function) (Table 1). Moreover, biological aging cells process in HF seems to start earlier in HF and can explain the morphological and functional changes in the HF syndrome as well as the higher prevalence of frailty condition in these patients [36].

More recently, a meta-analysis found greater frailty prevalence rates in cardiovascular disease, especially in patients with HF and aortic valve disease [37]. Notably, mortality rates in frail populations were 2.5 to 3.5 times higher compared to non-frail patients. Moreover, mortality rates of frail patients with HF were around 50% [37].

In conclusion, frailty is highly prevalent among CVD patients, in which frailty and CVD share common pathophysiological changes and severely affects clinical outcomes.

## 4. Frail Elderly Patients with ACS: A “Different” Population

Despite the increasing incidence of ACS with ageing, management of frail elderly patients undergoing ACS is still challenging since no standardized guidelines for conservative or invasive (i.e., percutaneous coronary intervention, PCI) treatment strategies are available.

In 2012, a retrospective analysis including 1001 patients aged over 75 years with STEMI or NSTEMI showed that cumulative survival for patients with interventional treatment was significantly better compared to the conservative treatment strategies (STEMI and NSTEMI *p* < 0.001) and these findings were confirmed in the same population sub-analysis including patients aged 75–85 years and in patients over 85 years old [38].

Shanmugam et al. [39] analyzed ACS 30-days mortality and re-infarction in very old patients (>80 years) with STEMI and NSTEMI, resulting in evidence in favor of a timely revascularization in contrast to conservative strategies (fibrinolysis, medical management). Hence, when frailty is prevalent in older ACS patient, rapid and full coronary revascularization seems the best option.

However, PCI is performed less frequently in frail elderly patients (frailty assessed by CHSA-CFS), probably because frailty is incorrectly believed to be associated with poor outcomes. In contrast, in frail elderly patients experiencing ACS, early reperfusion by PCI within 12 h decreased the in-hospital mortality even in ≥80 years frail patients with STEMI-ACS [40,41].

In patients undergoing ACS requiring percutaneous transluminal coronary angioplasty (PTCA), frailty ranged from 10% to 48% and higher level of frailty was associated to worse outcomes [42,43,44]. Ekerstad et al. [45] showed that frailty (assessed by CSHA-CFS) was independently associated with short-term outcomes for elderly patients with NSTE-ACS. In a cohort of NSTE-ACS patients from TRILOGY ACS trial, near 25% of patients were frail or pre-frail, confirming frailty being independently associated to increased cardiovascular death, myocardial infarction [46]. In the CONCORDANCE Registry, frail elderly patients experiencing ACS also showed higher all-cause in-hospital mortality (OR: 1.38, 95% CI: 1.05–1.83, *p* = 0.02) and higher 6 months all-cause mortality (OR:1.74, 95% CI: 1.37–2.22, *p* < 0.001), whereas cardiac specific in-hospital mortality and 6 months mortality were not significantly higher in frail vs. not frail patients [47].

Valvular heart disease (VHD) prevalence increases with age; 1 of 5 elderly patients (>74 years) experiencing hospitalization for ACS has a significant VHD including moderate to severe mitral regurgitation (MR), Aortic Stenosis (AS) or both [48]. Evidence of composite endpoint of all-cause death, myocardial infarction, disabling stroke and re-hospitalization for Heart Failure (MR:HR = 2.04, 95% CI: 1.36–3.07; *p* < 0.001; AS:HR = 3.10, 95% CI: 1.39–6.93; *p* < 0.01; both MR and AS:HR = 4.0 95% CI: 1.65–9.73; *p* < 0.001) and cardiovascular death (MR:HR = 3.17, 95% CI: 1.57–6.42; *p* < 0.01; AS and both MR and AS not significant) showed strong impact of VHD on elderly population prognosis [48]. High surgical risk in frail elderly patients often limited open heart valve replacement; conversely, mounting evidence showed benefits of trans-catheter aortic valve implantation (TAVI) compared to medical therapy [49,50]. After TAVI, frailty represents an independent predictor of major adverse cardiac events (HR: 4.2, 95% CI: 2.0–8.8) [51] and is associated with increased 1 year-mortality (HR: 3.5, 95% CI: 1.4–8.5; *p* = 0.007) [52]. Conversely, in elderly patients, no significant association between frailty status and periprocedural complications has been reported [52]. The FRAILTY-Aortic Valve Replacement (FRAILTY-AVR) study, including patients aged 70 to 99 years, showed that frailty is a major risk factor for death and disability after TAVI or surgical aortic valve replacement (SAVR); therefore, frailty should be accurately detected and treated in order to improve clinical outcomes [16,53,54]. Incidence of coronary artery disease (CAD) in TAVI population is between 40–75%. In elderly patients with CAD undergoing TAVI, frailty is an independent predictor of mortality and adverse events (HR: 2, 95% CI 1.38–2.89; *p* < 0.001) [55]. Notably, TAVI can be safely performed in patients asymptomatic for coronary ischemia without preoperative revascularization [55]. Post interventional cardiac rehabilitation showed benefits in patients independently after TAVI or SAVR on functional capacity and quality of life, but often limited from patient comorbidity, poor nutritional status and reduced mobility [56,57]. A pre-interventional rehabilitation programs aimed at improving elderly frail patients’ pre-interventional status and short and long-term prognosis has been proposed [58]. Notably, in patients referred to cardiac rehabilitation centers after TAVI, a significant improvement of disability index (Barthel Index 83.0 ± 21.2 vs. 62.1 ± 24.5, *p* < 0.001) and exercise capacity (6MWT distance 238.3 ± 76 vs. 175.6 ± 80 m, *p* < 0.001) both independent predictors of long-term 3-years mortality has been observed [59].

## 5. Frail Elderly Patients with ACS: The Dilemma of Balancing Atherothrombotic and Bleeding Risk

Antiplatelet therapy has been strongly recommended in patients experiencing ACS independently from age [60]; however, frail elderly patients’ management of antiaggregating drugs remains challenging due to age-related modifications of hemostatic balance [61]. In fact, major risk factors for major bleeding are represented by age, frailty condition and coexistence of several chronic pathological conditions: prior major bleeding, active malignancy, recent major surgery, lower blood hemoglobin levels, chronic kidney disease, liver cirrhosis, low platelet count and diabetes [62,63,64]. Despite high bleeding risk, in patients with pluri-comorbidity as diabetes, multivessel coronary artery disease, concomitant inflammatory disease (including infective disease as in the case of SARS-CoV-2 infection) and hematological status, atherothrombotic risk is considered moderate-higher [60,61,62,63,64,65,66,67].

In patients after ACS undergoing PCI, standard 12 months dual antiplatelet therapy (DAPT) (aspirin plus P2Y12-receptor inhibitors) is usually recommended [60] aiming at reducing 1-year atherothrombotic events [67]. Both DAPT duration and powerful of P2Y12-inhibitor can impact on bleeding risk [68]. Data from a meta-analysis exploring 10 trials (*n* = 32,287 patients undergoing PCI) showed that short DAPT, compared to standard 12 months therapy, significantly reduces major bleeding (OR: 0.58, 95% CI: 0.36–0.92) without increasing ischemic and thrombotic risk outcomes of myocardial infarction odds: 0.53 (0.42–0.66; *p* < 0.001) and stent thrombosis odds: 0.33 (0.21–0.51; *p* < 0.001), independent of P2Y12-inhibitor used [69]. A more recent meta-analysis including 52,816 patients with ACS exploring safety and efficacy of oral P2Y12 inhibitors (prasugrel, clopidogrel and ticagrelor) showed that ticagrelor significantly reduced cardiovascular mortality and all-cause mortality compared to clopidogrel (HR: 0.82, 95% CI: 0.72–0.92; HR: 0.83, 95% CI: 0.75–0.92), whereas no significant differences between prasugrel and clopidogrel (HR: 0.90, 95% CI: 0.80–1.01; HR: 0.92, 95% CI: 0.84–1.02) and between prasugrel and ticagrelor (HR: 1.10, 95% CI: 0.94–1.29 HR: 1.12, 95% CI: 0.98–1.28) have been reported [70]. Despite this evidence, major bleedings were significantly higher with prasugrel and ticagrelor compared to clopidogrel (HR: 1.26, 95% CI: 1.01–1.56; HR: 1.27, 95% CI: 1.04–1.55, respectively) [70].

Elderly population is often underrepresented in ACS trials and DAPT standard duration of 12 months has been largely discussed in recent years, especially in frail elderly population [68]. In fact, frailty has been associated to in-hospital bleeding [71] and predicts major bleeding within 30-day follow-up to discharge increasing all-cause mortality [72]. A specific trial on elderly patients undergoing PCI post-ACS and treated with DAPT showed no difference on primary outcome (composite of all-cause death, MI, disabling stroke, rehospitalization for cardiovascular causes and rehospitalization for bleeding) in those treated with clopidogrel vs. prasugrel at a reduced dose 5 mg instead of 10 mg/die [73].

Due to the increased risk of bleeding, a short DAPT has been proposed for frail elderly patients undergoing PCI after ACS. Risk/benefit of short DAPT has been largely discussed [74,75,76] and several scores have been proposed aiming at tailoring DAPT prescription in frail elderly patients. In particular, PRECISE-DAPT score (PREdicting bleeding Complications In patients undergoing Stent implantation and subsEquent Dual Anti Platelet Therapy) [77] for stratifying bleeding risk at 1-year after PCI intervention should be routinely assessed, even though in very low-risk patients, for better discriminating the intermediate bleeding risk category. In fact, in elderly patients (>74 years), PRECISE-DAPT showed more accuracy in stratifying patients at low/intermediate risk category (Table 1). The Consensus of Academic Research Consortium for High Bleeding Risk (ARC-HBR) defined criteria for bleeding outcomes in patients undergoing PCI [63]; however, the lack of data on frail elderly patients still limits its use in routine clinical practice [63].

In patients with high bleeding risk according to PRECISE-DAPT score [60], a short DAPT (1–3 months) and DAPT de-escalation after 1–3 months should be considered. Recent evidence suggests that ticagrelor on monotherapy after 3 months DAPT vs. 12 months ticagrelor-aspirin DAPT significantly reduced major bleeding outcome and cardiovascular events [78]. Subgroup analysis conducted in patients aged 75 years and older of the GLOBAL LEADERS trials showed that 1-month DAPT succeeded by 23 months ticagrelor monotherapy did not expose to higher risk of all-cause death or MI compared to 12 months DAPT followed by 12 months aspirin therapy. Notably, a lower trend for rate of stent thrombosis has been reported in patients treated with 1-month DAPT succeeded by 23 months ticagrelor monotherapy [79]. Interestingly, in the TWILIGHT study enrolling 9000 high-bleeding/ischemic risk patients undergoing PCI and completed 3 months of dual antiplatelet therapy, additional 12 months ticagrelor monotherapy was significantly associated to a lower rate of clinically relevant bleeding than ticagrelor plus aspirin, with no higher risk of death, myocardial infarction or stroke. [80].

Future trials are eagerly awaited aiming at exploring both the discriminating power of bleeding scores and the best DAPT strategies (i.e., duration and combination therapy) in frail elderly patients after ACS undergoing PCI.

## 6. Cardiac Rehabilitation after ACS in Frail Elderly Patients: Evidence and Goals

Despite that CR is widely recognized as a class 1A recommendation intervention [81,82], only 20% of eligible patients entered and completed 36 one-hour sessions of CR programs [83]. The benefits of exercise-based CR on several physiologic outcomes such as exercise capacity [84,85,86,87], myocardial flow reserve [88], autonomic function [89,90], lean tissue mass and function [91,92,93] (even in older patients) [94], blood pressure [95] and lipid profile are clearly demonstrated [94].

Sarcopenia, fatigue, exercise intolerance, cognitive decline, depression or worsened socio-economics factors, which are all hallmarks of frailty status, often limit the access of elderly patients to CR [96] (Table 1). Despite the proportion of frail elderly patients entering CR programs is growing, thanks to improvement and timeliness of ACS treatment, still few clinical trials investigated the benefits of CR in frail older patients. These patients present multiple comorbidities and/or disability at time of ACS presentation: it is mandatory developing individualized and tailored CR programs and define new treatment goals for this specific cohort [97].

Studies in normative elderly population showed that structured multicomponent exercise training based on a combination of strength and aerobic exercise program, plus balance and flexibility training, improve functional status, muscle function, mobility [98] and gait ability [99] and reduce fall risk and improve QoL, especially in oldest female and at early stages of frailty [100]. However, it is still uncertain whether these improvements may be obtained even in frail elderly patients in CR [101]. Notably, the EU-CaRE multicenter study aimed at comparing the intensity of CR training and peak oxygen consumption (peak VO_2_) changes associated to CR programs among European centers [102]. Interestingly, although participants underwent training sessions above anaerobic threshold (AT), peak VO_2_ had a greater improvement; however, a clear association between training intensity and peak VO_2_ improvement was not systematically detected, especially in elderly patients performing submaximal exercises (RER < 1.1) (i.e., half of study cohort) [103].

Despite evidence of multicomponent exercise training benefits on muscle strength, gait speed, balance and physical performance in elderly frail and pre-frail patients, type, duration and intensity of exercise prescription in these patients are not completely standardized. Exercise training should be adapted and increased based on single patient physical capacity, trying to reach autonomy and independence in activity of daily living [104]. In clinical practice, exercise training (when feasible) should start with more accessible and safe exercise and increase gradually, to prevent symptoms or complications: frequency of sessions should be 2–3 times a week, strength exercises for 40 to 60 min, including resistance training, static and dynamic balance and flexibility exercise. In frail patients unable to perform CPET, during training sessions, heart rate (HR) should be set at slightly lower than HR achieved in 6MWT and carefully monitored during exercise sessions. Clinical supervision is suggested in order to avoid discomfort and to detect fatigue or other symptoms [105].

High intensity interval training (HIIT) achieves the best functional results in cardiac rehabilitation, but, due to safety concerns in patients at high risk of adverse events, a moderate-intensity interval training (MIIT) represents an alternative in elderly or frail patients [106,107,108,109].

For elderly frail patients, Dun et al. [95] proposed short-interval HIIT, consisting of high-intensity interposed by low-intensity exercise sessions. Patients with lower functional capacity can start with this kind of exercise and progressively increase time in high intensity exercise according to single exercise adaptation during the weeks of CR [110].

Therefore, in frail elderly patients, clinical intervention must focus on physical efficiency assessed by aerobic capacity (Cardiopulmonary exercise test, 6 min walking test), on functional autonomy (ADL) and on improvement of muscular strength, balance and flexibility (for which the Short Physical Performance Battery has showed great sensibility). Tailored CR programs based on individual functional level can help to manage the complexity of the older and most frail patients [111].

## 7. Management of Frail Patients: A New Challenging Model of Care for CR Community

Mean age of patients entering CR programs is increasing and, consequently, the presence and degree of frailty, number of comorbidity and disability influence setting, type and intensity of exercise training programs. Frail and pre-frail patients gain benefits from CR improving functional autonomy, quality of life as well as in reducing the cardiovascular risk. Despite evidence, there is a lack of guidelines of elderly patient’s management. Management protocols must be developed to identify frail and pre-frail patients at early stages, when clinical interventions based on personalized care may reverse frailty process. New CR programs should be hybrid (both hospital- and home-based) and individualized for frail patients with cardiovascular disease [112,113].

Home-based CR (HBCR) exercise setting represents a validate alternative to center-based CR (CBCR) in patients with logistical problems or lack of socioeconomic support. All core components overlap with center-based CR: exercise training, risk factors management (dietary education, smoking cessation) medication management and psychological support; majority of HBCR exercise training consists of a 4-week (12 session) center based monitored with telemetry and 4 weeks (12 sessions) home based supported by video call and physiotherapist or nurse presence. Goals of HBCR in older adults is preserving functional capacity and independence, preventing onset of disability; HBCR seems instead to have a role in falls prevention, in ambulation maintaining, in muscular strength and quality of life improving [93]. Hospital-based and home-based Cardiac Rehabilitation in 14,486 patients, included in recent meta-analysis with 12 months follow-up, both reduced re-hospitalization and cardiovascular mortality and improved quality of life, in general CAD patients [94], but the evidence that home-based cardiac rehabilitation is safe and effective in frail elderly is still insufficient. No difference in peak oxygen uptake and 6 min walking distance outcomes have been reported between HBCR and CBCR. In addition, similar positive results have been obtained for risk factor modification (weight, smoking cessation, blood pressure, lipid profile). However, no statistical differences in all-cause mortality were found between HBCR and CBCR at 12 moths follow-up [114].

Most of the frail elderly patients eligible for CR have limitations to access the HBCR programs: the patients need caregiver-support to use mobile technologies and physiotherapists to correctly perform exercises. However, safety of HBCR has not completely explored especially for the very old population and for high-intensity training programs [115]. Telemedicine may also be an additional instrument for frail elderly who cannot attend hospital-based CR. Pilot studies and reviews has been recently conducted to report benefits of HBCR using remote supervision and telerehabilitation platforms: in HF elderly patients’ improvement in 6MWT distance after 12-week CR was statistically significant [116]. In patients experiencing HBCR vs. CBCR, no difference in outcome of mortality [117], functional capacity and HRQL has been reported [118], not statistically difference has been found in maximal aerobic exercise capacity assessed by peak oxygen consumption; however, adherence to exercise program remains higher in HBCR [119]. During COVID-19 pandemic, telerehabilitation take the scene as effective alternative, paving the way for reallocating resources in telehealth for frail elderly patients for which is not desirable attending hospital centers [120]. However, the safety and applicability of these tools in frail elderly population is still uncertain and benefits have not been fully elucidated.

## 8. Conclusions

Frailty represents one of the major challenges for cardiac rehabilitation community. Frail patients with acute or chronic coronary heart disease are often denied procedures or multidisciplinary exercise-based cardiac rehabilitation programs. Future studies are eagerly awaited in order to identifying the best tool for frailty assessment for developing individualized models of care. Since the population of patients aged 75 or older is growing, frailty is going to be an emergent social and medical issue. Home-based cardiac rehabilitation programs and telerehabilitation models specifically designed for elderly frail patients are eagerly encouraged. Once frailty in cardiac elderly patients is detected, exercise is the best therapeutic strategy to reverse or mitigate frailty, preserve quality of life and restore independent functioning in older adults.

## Figures and Tables

**Table 1:** Principal domains, components and tools for frailty patients with cardiovascular disease enrolled to Cardiac Rehabilitation programs.

Domains	Components	Tools	Interpretation and/or Norm Values	Interventions
Functional Capacity	Exercise capacity	Muscle fatigue, Gait Speed, 6MWT	Gait speed > 0.8 m/s: 6MWT must re-performed in time (patient itself control)	Endurance and/or resistance training
	Muscle strenght	Handgrip	Handgrip (Fried’s criteria): Men ≥ 29–32 kgf and Women ≥ 17–21 kgf (adjusted for BMI)	Resistance training
	Balance	Tinetti scale/SPPB	Fall risk is moderate to high if SPPB < 10 and Tinetti scale < 25	Balance and coordination training
	Sarcopenia	Gait speed/SPPB	Gait speed > 0.8 m/s, Handgrip (Fried’s criteria): Men ≥ 29–32 kgf and Women ≥ 17–21 kgf (adjusted for BMI), SPPB < 10	Endurance walking exercise
	Energy expenditure	MLTAQ-short version	Kcal/week expended: Men < 383 Women < 270	Reduce sedentary time and increase daily-life activity
	Dependency	ADL/IADL	Reach independence: ≥4/6 items, ≥6/8 items	Provide functional exercises relevant to daily life
Comorbidity	Number and severity of chronic conditions	Medical record analysis, CIRS	Check for major (chronic) conditions	Tailor intervention based on medical history
	Bleeding risk	HAS-BLED, PRECISE-DAPT score	HAS-BLED 5–8 High risk; PRECISE-DAPT score ≥ 25 Short DAPT	Optimize therapy: Standard/long DAPT (12–24 months), short DAPT (3–6 months)
	Polypharmacy	Teraphy check	Number/ indication of single drug	Avoid adverse combinations and/or reduce unnecessary medications
	Anemia	Laboratory testing	Serum Hemoglobin level: Men >13 g/dL Women >12 g/dL (check MCV, iron blood levels, serum ferritin, TIBC, Vitamin B12, folate)	Consider iron prescription/erythropoetin
	Albuminemia	Laboratory testing	Serum Albumin 3.5–4.5 mg/dL	Nutritional counseling
	Diabetes	Laboratory testing and clinical examination	Fasting plasma glucose >100 mg/dL and HbA1c <6.0%; Check diabetes neuropathy, retinopathy	Elevate physical activity, health nutrition with weight loss and/or prescription of metformin
	Dyslipidemia	Laboratory testing	Total Cholesterol, LDL cholesterol, TG, norm value dependent on CV risk	Elevate physical activity, health nutrition with weight loss and/or prescription of statin
	Thyroid dysfunction	Laboratory testing	TSH, FT3, FT4: normal value dependent on age and sex	Check periodically
	Renal dysfunction	Laboratory testing	Blood Urea Nitrogen, Creatinine, eGFR, normal value dependent on age and sex	Check periodically
	Liver dysfunction	Laboratory testing	AST, ALT, GGT, normal value dependent on age and sex	Check periodically
	Hypovitaminosis	Laboratory testing	25-OH-vitaminD (>30 ng/mL)	Consider prescription of 25-OH-vitaminD or Calcifediol
	Disturbed blood pressure	BP assessment	Systolic blood pressure target in elderly no lower than 130 mmHg	Consider pharmacotherapy in case of hypotension or hypertension
	Hearing loss	Audiometry	Impaired hearing function, especially conversational frequencies	Consider hearing aids
	Pulmonary dysfunction	Spirometry	Check FEV and Tiffenau index	Consider specialist counseling
	Cardiac dysfunction	Ecocardiography/Laboratory testing	Ejection fraction % ≥ 55, E/A ratio ≥ 1, NT-proBNP < 450 pg/mL (in 75–99 years)	Optimize therapy
Nutritional status	Calories intake	MNA	Check for adequate calorie intake	Promote sufficient calories intake
	Protein intake	NRS 2002	Check for adequate protein intake: in elderly around 1–1.2 g/kg/day	Promote healthy protein-rich food items
	Loss of appetite	Self-reported unexplained weight loss	More than 4.5 kg or 5% of body weight in past year	Promote sufficient calories and protein intake
	Water intake	Dehydratation	Check for water intake	Promote water intake adequate on singular needing
Cognitive Function	Memory and executive functions	MMSE	Detect Mild Cognitive Impairment (MCI): MMSE < 26 points (age and scholar correction)	Consider specific diagnostic study
Physicological function	Mood	GDS	Check for mood disorders if GDS < 10 points	Consider specific diagnostic study
	Cognition	MINI-Cog test	Score ≥ 3 indicate lower likelihood of dementia	Consider specific diagnostic study
Social Support	Family or community support	Caregiver presence	Check for family support	Help organising faimily support
	Financial resources		Check for financial issues/constraints	Refer to social worker if needed
	Smoking behaviour	Anamnesis	Avoid active and passive smoking	Quit smoking and/or avoid smoking exposure

**Captions**: 6MWT, 6 min walking test; ADL/IADL, Activities of daily living/Instrumental activities of daily living; AST, Aspartate Aminotransferase; ALT, Alanine Aminotransferase; BMI, Body mass index; BP, Blood Pressure; CIRS, Cumulative Illness Rating Scale; CPET, Cardiopulmonary exercise test; CV risk, Cardiovascular risk; DAPT, Dual Antiplatelet Therapy; eGFR, Estimated glomerular filtration rate; FEV, Forced Expiratory Volume; GGT, Gamma-Glutamyl Transferase; GDS, Geriatric depression scale; HASBLED score, Hypertension, Abnormal renal/liver function, Stroke, Bleeding history, Labile International Normalized Ratio or INR, Elderly >65 years, Drugs/alcohol concomitantly; HbA1c, Glycated Hemoglobin; LDL cholesterol, Low Density Lipoprotein; MCV, Mean Corpuscular volume; MLTAQ-short version, Minnesota Leisure Time Activity Questionnaire; MMSE, Mini mental state examination; MNA, Mini nutritional assessment; NRS 2002, Nutritional risk screening; NT-pro-BNP, N terminal pro-brain natriuretic peptide; PRECISE-DAPT, PREdicting bleeding Complications In patients undergoing Stent implantation and subsEquent DAPT; SPPB, Short physical performance battery; TG, Triglycerides; TIBC, total iron binding capacity; FT3, Free Triiodothyronine; FT4, Free Thyroxine; TSH, Thyroid-stimulating hormone.

## Data Availability

Not applicable.
